# Teaching programming and computational thinking in early childhood education: a case study of content knowledge and pedagogical knowledge

**DOI:** 10.3389/fpsyg.2023.1252718

**Published:** 2023-10-02

**Authors:** Yue Zeng, Weipeng Yang, Alfredo Bautista

**Affiliations:** ^1^School of Education, Wenzhou University, Wenzhou, China; ^2^Department of Early Childhood Education, The Education University of Hong Kong, Hong Kong, Hong Kong SAR, China

**Keywords:** programming, computational thinking, early childhood teacher, content knowledge, pedagogical knowledge

## Abstract

Programming and computational thinking (CT) have been progressively incorporated into early childhood education to prepare children for the digital age. However, little is known about the content knowledge (CK) and pedagogical knowledge (PK) possessed by early childhood teachers in this domain. To address this gap, we conducted a case study of an early childhood teacher in China who had experience developing and implementing an unplugged programming and CT curriculum. The triangulation of data sources was established to collect evidence from videotaped observations, interviews, and lesson plans. For the CK, analysis of these findings revealed that the teacher had a more robust understanding of CT concepts (e.g., sequences, conditionals, and loops) compared to CT practices (e.g., decomposition, debugging) and CT perspectives (e.g., perseverance, choices of conduct). In terms of PK, the teacher could apply the general pedagogical knowledge but was relatively weak in using content-specific pedagogical knowledge. As the first endeavor to investigate an early childhood teacher’s CK and PK in teaching programming and CT, this study provides significant implications for improving teachers’ professional knowledge and teaching effectiveness in this burgeoning area.

## Introduction

Globally, an increasing focus has been placed on teaching programming and computational thinking (CT) in early childhood education (ECE) (Bers et al., 2022; Yang et al., 2023). CT, viewed as a core competency in the 21^st^ century, is related to solving problems that are often open-ended and complex in various disciplines with the use of the concepts fundamental to computer science (Wing, 2006). CT involves the ability to break down complex problems into smaller parts, identify similarities among and within problems, develop step-by-step solutions and so on (Yang et al., 2022; Zeng et al., 2023). Programming, on the other hand, is the process of writing codes to implement a particular task or solve a particular problem (Mills et al., 2021). CT and programming are closely intertwined, with each relying on and enhancing the other. Programming necessitates CT skills to create efficient and effective code (Lye and Koh, 2014), while programming plays a crucial role in the development of CT (Voogt et al., 2015). For example, when programming, a programmer often needs to break down a complex task into smaller parts, recognize patterns in data, and identify the most efficient approach for each step. This process involves CT skills such as pattern recognition, algorithmic thinking, and abstraction, which can then be applied to other domains, such as mathematics, science, and engineering.

Teachers’ pedagogical content knowledge (PCK), which represents the incorporation of content and pedagogy into an understanding of how to make the teaching content easily understood by students with diverse abilities and interests (Shulman, 1987), is critical in predicting and enhancing young children’s learning in domain-specific areas (Dunekacke and Barenthien, 2021). Previous research indicated that providing support for teachers’ PCK had a positive impact on their teaching practices and children’s development (Gözüm et al., 2022). However, few studies have examined early childhood teachers’ PCK for teaching programming and CT. To fill this gap, this study aims to investigate early childhood teachers’ content knowledge (CK) and pedagogical knowledge (PK) in teaching programming and CT. Specifically, we employed two frameworks to analyze an early childhood teacher’s CK and PK that is demonstrated in planning, implementing, and reflecting on programming and CT activities. This investigation is crucial for providing training that focuses on addressing the areas of weak CK and PK among early childhood teachers, thus enhancing the effectiveness of teaching in early programming and CT.

### Previous studies on unplugged programming and CT education

Programming and CT education is primarily conducted through two approaches: the plugged approach and the unplugged approach. The plugged approach involves using digital devices such as tablets, computers, and the Internet. In contrast, the unplugged approach aims to teach programming and CT without any digital devices, instead utilizing materials like pen and paper, cards, or engaging in physical activities (Otterborn et al., 2020). Romero et al. (2018) summarized the key benefits of the unplugged approach, including embodied learning, reduced cognitive load, and concrete analogies. The unplugged approach often incorporates physical actions and tangible manipulation, aligning well with the learning styles of young children. Furthermore, compared to digital tools, incorporating unplugged materials in programming and CT education could minimize distractions that divert children’s attention and reduce cognitive load, which refers to information-processing (attentional or working-memory) demands (Block et al., 2010). Lastly, unplugged activities are built upon the construction of tangible and concrete analogies, facilitating the learning of abstract concepts related to programming and CT. Several studies have explored the effectiveness of the unplugged approach in promoting learners’ CT (del Olmo-Muñoz et al., 2020; Saxena et al., 2020; Ahn et al., 2021; Li and Yang, 2023). In this study, the way the teacher employed to teach programming and CT is the unplugged approach.

### The content framework of computational thinking in ECE

The goal of early programming and CT education is not to prepare children to become programmers or algorithmic engineers but rather to foster their CT. As argued by Resnick and Robinson (2017), children do not simply “Learn to Code” but rather “Code to Learn” and “Learn Through Coding.” Thus, our interest lies in identifying the core content of CT covered and emphasized in early childhood teachers’ instruction of programming and CT. To achieve this, we reviewed the CT content framework in ECE.

There is a lack of a consistent content framework for CT in ECE (Zhang and Nouri, 2019). After comparing different CT frameworks, Zeng et al. (2023) used Brennan and Resnick’s (2012) three-dimensional CT framework to identify CT components that were proven appropriate for young children to learn and established the CT curriculum framework for ECE. This framework articulates the core content in early programming and CT education, covers CT concepts (i.e., control flow/structures, representation, and hardware/software), CT practices (i.e., algorithmic design, pattern recognition, abstraction, debugging, decomposition, iteration, and generalizing), and CT perspectives (i.e., expressing and creating, connecting, perseverance, and choices of conduct) (Zeng et al., 2023) (see Table 1).

**Table 1:** The CT content knowledge framework in ECE (Zeng et al., 2023).

CT dimensions	CT components	Descriptions
CT concepts	Sequences	A specific task or activity is conveyed as a succession of separate commands or steps that a human or machine can carry out (Brennan and Resnick, 2012)
	Loops	A mechanism of repeatedly executing the same instructions (Brennan and Resnick, 2012)
	Conditionals	Allowing for the expression of different outcomes by making decisions based on certain circumstances (Brennan and Resnick, 2012)
	Events	“One thing causing another thing to happen” (Brennan and Resnick, 2012, p. 4)
	Representation	In programming, representation refers to the use of symbols to represent instructions (Bers, 2018)
	Hardware/ Software	Hardware and software operate in tandem to complete tasks; the software gives the hardware instructions, and the hardware executes those instructions (Bers, 2018)
CT practices	Algorithmic design	A set of sequential, organized steps used to solve a problem or complete a task (Bers, 2018)
	Pattern recognition	Identifying patterns and trends (commonalities) between and within problems to simplify the solution (Hsu et al., 2018)
	Abstraction	The conscious effort to ignore irrelevant details and focus only on the important information, thus making problem solving easier (Lee et al., 2022)
	Debugging	Identifying and repairing mistakes when solutions do not work as expected (Wang et al., 2020)
	Decomposition	Breaking down a complex problem or system into smaller easily solved or managed parts (Wing, 2011)
	Iteration	Seeking upgrades of solutions using design processes repeatedly until the optimum solution is obtained (Shute et al., 2017)
	Generalizing	Transferring approaches used to address particular issues to new situations (CSTA and ISTE, 2011)
CT perspectives	Expressing and creating	Seeing computation as a way for designing and conveying ideas (Brennan and Resnick, 2012).
	Connecting	Cooperating, communicating with others and sharing works with others (Brennan and Resnick, 2012)
	Perseverance	Persevering in the face of challenges or failures and seeing failures as usual to reach a goal (Wang et al., 2020)
	Choices of conduct	Deciding what to do and what not to do in a specific situation by oneself (Pugnali et al., 2017)

### Pedagogical issues related to teaching programming and CT in ECE

This section summarizes the teaching context, activity structure, pedagogical approaches, and pedagogical strategies previously used to foster children’s programming and CT skills (see Table 2).

**Table 2:** The programming and CT pedagogical knowledge framework in ECE.

Dimensions	Indicators	Description
Teaching context	Group activity	Purposeful, planned activities organized by the teacher in which many children in the class participate
	Learning center	Different learning areas in the classroom self-chosen and -directed by children
	Daily lives and routines	Children’s daily lives and routines such as having meals, washing hands, and tidy up toys
	Integrative learning contexts	Connecting programming and CT with other learning domains such as art, math and literacy
Activity structure	Highly structured	Objectives pre-defined by teachers, and the activities primarily initiated by teachers
	Open-ended	Activities that allow children to freely explore
	Mixed	Activities that include both structured activities and open-ended activities and/or free play (Bakala et al., 2021)
Pedagogical approaches	Task-based learning	Teacher-directed pedagogical approach in which learning activities are organized around adult-guided tasks (McCormick and Hall, 2021)
	Project-based learning	Activities that allow children to explore relatively independently for long periods and yield real works or presentations (Kokotsaki et al., 2016)
	Problem-solving learning environment	A learning environment proposed by Lye and Koh (2014) that can enhance students’ CT practices and perspectives, which include authentic problem, information processing, scaffolding and reflection
	Play-based learning	A playful, child-directed pedagogical approach with some adult direction and learning goals (Pyle and Danniels, 2017)
	Others	Other pedagogical approaches not covered in this list
Pedagogical strategies	Unplugged activity	Learning programming and CT without a computer and is often conducted through bodily activity or with other learning materials (Otterborn et al., 2020)
	Embodied cognition	Using embodied activities to help children understand abstract CT concepts (Moore et al., 2020; Saxena et al., 2020)
	External memory support scaffolding	Providing supplementary materials to turn abstract algorithms into visible and concrete representations to help children cope with working memory limitations and reduce cognitive load (Macrides et al., 2021)
	Pair programming	A collaborative programming approach in which two students work together to complete the same programming task (Denner et al., 2014)
	Differentiated Instruction	Providing children with appropriate scaffolding based on each child’s individual abilities and needs (Wang et al., 2020)
	Demonstration	Modeling the necessary skills and attitudes to children (Wang et al., 2020)
	Others	Other pedagogical strategies not covered in this list

#### Teaching context

Lee and Junoh (2019) noted the importance of infusing programming and CT into children’s daily lives and setting up programming centers/corners in early childhood classrooms. Mills et al. (2021) emphasized that integrating programming and CT into other learning domains would provide meaningful learning contexts for young children.

#### Activity structure

There are three categories of programming and CT activity structure: *highly structured, mixed, and open-ended*. Most studies designed highly structured programming and CT activities (Nam et al., 2019; Khoo, 2020) and few studies designed open-ended free play with programming tools. Newhouse et al. (2017) found that the children appeared more engaged and motivated in the high teacher-supported sessions rather than in free play without explicit scaffolding. Other studies designed mixed activities (Bers et al., 2014, 2019; Strawhacker and Bers, 2019). For instance, in the study by Strawhacker and Bers (2015), there was always a “buffer lesson” for children to explore the programming materials freely, which allowed them to absorb what they had learned and kept their attention throughout other highly structured activities.

#### Pedagogical approaches

Early programming and CT education employs a variety of pedagogical approaches. One such approach is the task-based approach, where learning activities revolve around tasks guided by adults (McCormick and Hall, 2021). Bers (2019) showed how such intentionally structured activities can aid young children in developing CT skills. Another notable approach is the project-based learning, characterized by its student-centered nature. This approach emphasizes students’ autonomy, goal-setting, planning, exploration, cooperation, and reflection within authentic real-world practices (Kokotsaki et al., 2016). Several studies involved activities of the construction of robots, engaging students in design, problem-solving, decision-making, and investigative tasks (Macrides et al., 2021). Play-based learning, on the other hand, presents a playful and child-directed pedagogical approach with some adult guidance and predefined learning objectives (Pyle and Danniels, 2017). Critten et al. (2022) suggested play-based, pedagogic practices can be used with children as young as 2 years to learn many of the basic concepts involved in CT skills. Moreover, Lye and Koh (2014) suggested designing a problem-solving learning environment, which includes authentic problems, information processing, scaffolding and reflection, to enhance students’ CT practices and perspectives.

#### Pedagogical strategies

Previous studies have examined the effectiveness of different pedagogical strategies for improving young children’s CT, including unplugged activities, embodied cognition, external memory support scaffolding, and pair programming. Unplugged programming uses materials like paper, cards, and blocks and has been shown to improve CT skills through embodied learning, lower cognitive load, and concrete analogies (Romero et al., 2018; Otterborn et al., 2020). While for embodied cognition, there are two kinds of embodiment according to the source of body movement: direct embodiment, which refers to moving bodies to perform solution steps; and surrogate embodiment, which refers to manipulating an external surrogate without engaging their bodies (Fadjo, 2012). External memory support scaffolding is used to help children cope with working memory limitations and reduce cognitive load during programming (Angeli and Valanides, 2020). Pair programming, a collaborative programming approach in which two students work together on a single computer to complete the same programming task, positively improved students’ programming and CT skills, learning motivation, metacognition, and collaboration (Denner et al., 2014; Papadakis, 2018). Besides these experimental studies, Wang et al. (2020) video observed various strategies an exemplary teacher used to support preschoolers’ CT skills, such as modeling a positive attitude toward error, breaking down problems into small steps, and providing different scaffolds according to children’s individual needs.

However, previous studies were mainly aimed at validating the effectiveness of a particular pedagogical strategy in improving children’s CT without examining what pedagogical strategies teachers used. Only Wang et al. (2020) investigated the pedagogical strategies used by a male teacher; however, this case study was conducted in a higher teacher-student ratio (1,3) context instead of a large-group context which is common in Asian cultural contexts.

### The PCK theory

**Figure 1:**
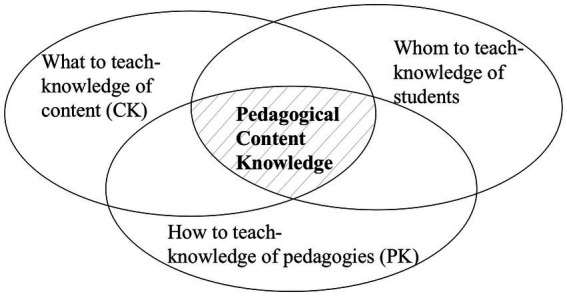
Pedagogical Content Knowledge (PCK) (McCray & Chen, 2012, redrawn).

PCK was first introduced by Shulman to emphasize the fundamental role of subject matter in (research in) teacher education and teaching in 1985. In subsequent years, PCK has been defined by different researchers in multiple ways. Despite the various definitions, researchers have identified three essential components of PCK: CK, PK, and knowledge of students’ understanding (Rojas, 2008; McCray and Chen, 2012; Zhang, 2015). Figure 1 illustrates how these three components are interrelated to the construct of PCK (McCray and Chen, 2012). This study specifically examined teachers’ CK and PK of programming and CT.

CK is the knowledge of what to teach. It encompasses knowledge of the discipline to be taught, a thorough understanding of that knowledge, and an understanding of the relationships between topics of the discipline (Krauss et al., 2008). In this study, we focused specifically on the first two aspects, i.e., whether the teachers knew the programming and CT knowledge to be taught and whether teachers had a deep understanding of them.

PK is the knowledge of how to teach. There are two types of PK: general pedagogical knowledge (GPK) and content-specific pedagogical knowledge (CPK). GPK comprises comprehension of various educational philosophies and learning theories, general knowledge of learners and basic teaching rules, and familiarity with classroom management principles and strategies (Grossman and Richert, 1988). CPK is the knowledge of instructional strategies unique to a particular subject or topic (Zhang, 2015). In this study, we examined both the GPK and CPK.

### Teachers’ PCK of programming and CT

Given the scant existing literature in this field, we conducted a comprehensive review focusing on the PCK of both preservice and in-service teachers across all educational levels. Several researchers have discovered that both pre-service and in-service teachers possess limited knowledge of CT and little knowledge of how to teach programming and CT (Bower and Falkner, 2015; Chalmers, 2018; Sands et al., 2018).

Accordingly, it has been suggested by researchers that there is a pressing need to enhance teachers’ PCK through pre-service and in-service training programs to facilitate the integration of CT into their classrooms (Yadav et al., 2017; Chalmers, 2018; Haines et al., 2019). Chalmers (2018) specifically emphasized that a deeper understanding of CT concepts, practices, and perspectives is crucial for teachers to effectively incorporate CT into the primary curriculum. Çakıroğlu and Kiliç (2020) proposed a course model and evaluation tools aimed at improving teachers’ PCK for teaching CT via robotic programming.

Within the context of ECE, Strawhacker et al. (2018) found that teachers who possessed a solid foundation of CK exhibited more purposeful use of the programming tool and gave more explicit support. Similarly, Wang et al. (2020) found that the case teacher intentionally employed various strategies in his programming and CT instruction because of his clear understanding of CT skills that young children need to develop.

### The present study

Previous research has indicated a need to improve teachers’ PCK through training to help them implement programming and CT education (Yadav et al., 2017; Chalmers, 2018; Haines et al., 2019). To provide targeted training to help teachers acquire the necessary PCK and effectively deliver programming and CT education, it is crucial to clearly understand the status of teachers’ PCK in programming and CT education. However, based on our thorough review of the existing literature, there is a lack of research specifically examining the status of CK and PK of programming and CT among early childhood teachers. As teachers’ CK and PK can be demonstrated in their teaching (Zhang, 2015), to examine early childhood teachers’ CK and PK, we proposed the following questions:

*RQ1*: What CT concepts, practices and perspectives were covered and emphasized in the early childhood teacher’s teaching of programming and CT?

*RQ2*: How did the early childhood teacher support children’s programming and CT learning?

## Methods

We employed a case study method, which allows people to gain a greater insight into a specific case by investigating it in depth and within its actual context (Yin, 2009). Our case study examined an early childhood teacher’s CK and PK in teaching programming and CT.

### The research site

This study was conducted in a provincial first-class public kindergarten located in Wenzhou, China, with a specific focus on STEM education. Recognizing the increasing importance of programming and CT education, the kindergarten embarked on a new educational initiative to integrate programming and CT into its curriculum. As an initial step, instead of implementing programming and CT education across all classes, the kindergarten decided to initiate a pilot program. They selected one class from each of the age groups: K1 (3-year-olds), K2 (4-year-olds), and K3 (5-year-olds), led by one teacher in each class.

We chose the K3 class for observation because the teaching content of the unplugged curriculum in the K3 class was built upon that of K1 and K2 and covered all the CT skills of the unplugged curriculum, thus allowing us to examine RQ1 comprehensively. The K3 class consisted of 32 children aged 5–6 years, along with two teachers and a nurse. For the purposes of this study, we selected Ms. Wu, who was responsible for teaching programming and CT in this class and who enthusiastically volunteered to join our study.

Initially, the three experimental classes utilized a plugged-in programming tool named MOBLO. MOBLO is a hybrid kit that enables young children to program a virtual character on the screen by manipulating tangible programming blocks. Due to concerns regarding the potential damage of screen usage on children’s eyesight, the kindergarten developed an unplugged, screen-free programming tool and an unplugged programming curriculum, taking inspiration from MOBLO. Subsequently, the three experimental classes conducted the unplugged programming curriculum. Notably, Ms. Wu not only implemented the unplugged curriculum but also participated in designing the unplugged programming tool and the unplugged curriculum.

Ms. Wu possessed 11 years of work experience in the field of ECE. She initially graduated from a local normal university with an Associate’s degree in ECE. Following 7 years of work experience, she pursued a Bachelor’s degree in ECE through adult correspondence education. However, neither of these programs included any courses related to early programming or CT education. Ms. Wu’s exposure to programming education came exclusively from the MOBLO company. To implement programming education with the MOBLO programming tool in the kindergarten, the MOBLO company provided training to teachers. This training primarily focused on instructing teachers on the utilization of the MOBLO programming tool and how to teach programming using the lesson plans provided by the MOBLO company.

**Figure 2:**
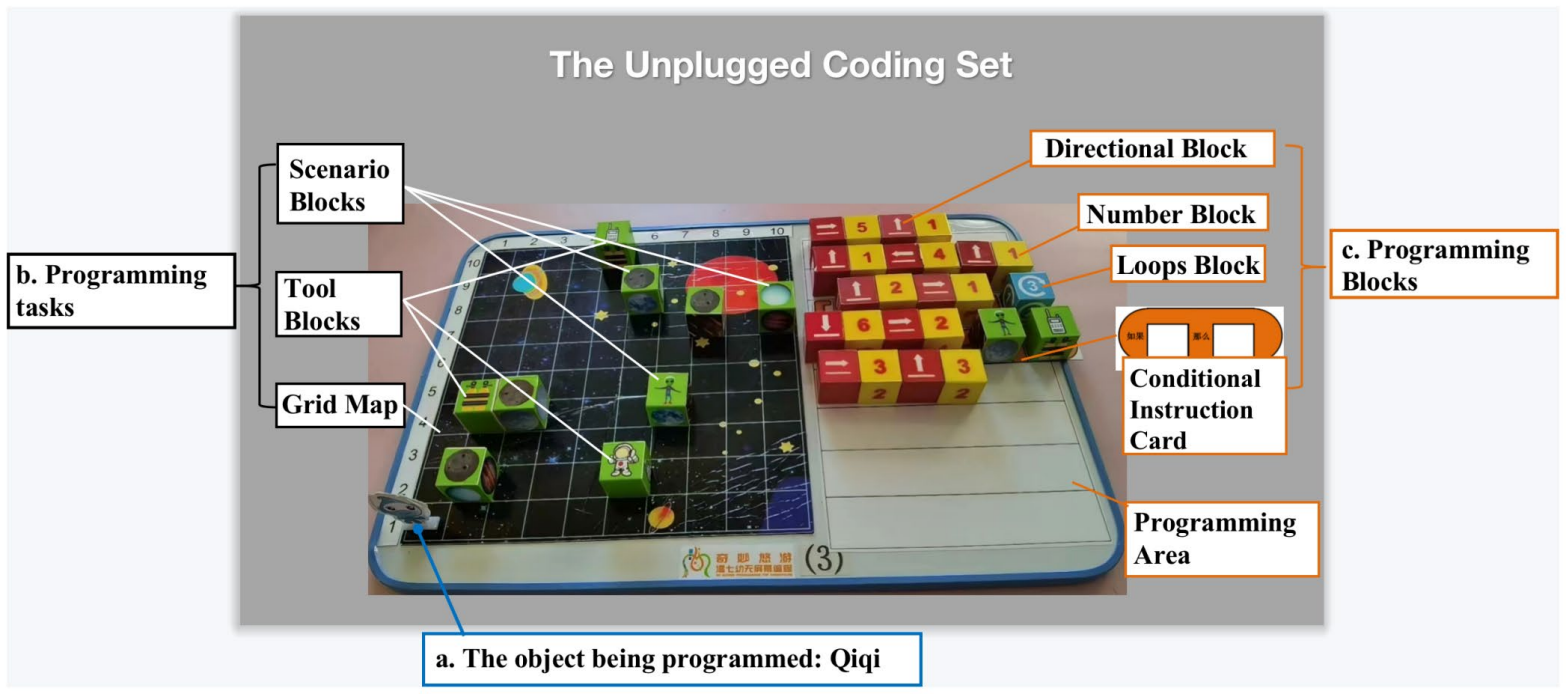
The Unplugged Coding Set.

The unplugged programming tool developed by this kindergarten, like other coding sets, consists of three parts: (1) The object being programmed: The object being programmed in this coding set is a pawn named Qiqi, who is also the protagonist of the stories in the unplugged curriculum; (2) Programming tasks: The teacher or children set up programming tasks by putting the Tool Blocks (representing tools Qiqi needs to obtain to solve problems) and Scenario Blocks (representing the characters, place, and things that happen in the story) on a 10 by 10 Grid Map. (3) Programming blocks: Children program the routes Qiqi takes by placing wooden Programming Blocks (including Directional Blocks, Number Blocks, Loops Block, and Conditional Instruction Card) in the Programming Area. For example, in the context of exploring outer space, Qiqi first needs to collect tools such as the spacesuit, oxygen kit, and translator. On his journey to other planets, he must avoid meteorites. When encountering problems, he needs to use tools (for example, using a translator when meeting an alien). Eventually, he reaches other planets (see Figure 2). Appendix 3 shows how to make a similar coding set using readily available materials.

### Data collection

As teachers’ PCK can be demonstrated in planning, implementing and reflecting on teaching (Zhang, 2015), lesson plans, videotaped programming activities, and audiotaped interviews were collected as our data to establish triangulation (Yin, 2009), as well as memos following each observation and interview.

#### Video observations

Compared to other data types, video has definite advantages in capturing the teaching content and pedagogies in classrooms (Jacobs et al., 1999). In conducting the video observation, two cameras were used, one was set in the corner of the classroom to ensure the whole class activities were recorded, and the other was held by the researcher to capture Ms. Wu’s interaction with children. A total of 12 lessons, each lasting approximately 40 min, over 6 weeks were video recorded, resulting in 728 min of video.

#### Interviews

We developed an interview protocol that focused mainly on two themes (in addition to a set of background questions): (1) Content Knowledge: Core content covered in the programming and CT course and the early childhood teacher’s understanding of them. (2) Pedagogical Knowledge: Pedagogical practices about supporting children’s programming and CT learning (including a focus on the teaching context, activity structure, pedagogical approaches, and pedagogical strategies), as well as the reasons for adopting these pedagogical practices.

We conducted both formal and informal interviews. The formal interview was conducted after all sessions to understand Ms. Wu’s CK and PK in early programming and CT (the interview protocol, see Appendix 2). It lasted around an hour. Informal interviews were conducted after class (if necessary) to have a deeper understanding of what had been observed.

#### Lesson plans

This study used lesson plans to supplement the observational and interview data. We collected a total of 12 lesson plans from Ms. Wu.

### Data analysis

To analyze the CT concepts, practices and perspectives that are covered and emphasized in the early childhood teacher’s teaching of programming and CT, we used the CT curriculum framework for ECE (Zeng et al., 2023), which has a detailed and clear definition of each CT component, as the CK Framework (see Table 1). The CK Framework includes three dimensions: CT concepts, practices, and perspectives.

To examine how the early childhood teacher supported children’s programming and CT learning, we developed the PK Framework (see Table 2). The PK Framework comprises four dimensions: teaching context, activity structure, pedagogical approaches, and pedagogical strategies. We constructed the indicators under each dimension based on the aforementioned literature review. Moreover, we provided a clear definition for each indicator in the PK framework (see Table 2).

Then the first author and the second author used the two frameworks to analyze the video data, the interview data, and the lesson plans. The following explains the process of data analysis. Appendix 1 shows a few examples of data analysis.

#### Video and interview data analysis

We analyzed recorded videos and interviews with the following steps:

Step 1. Transcription of selective video clips and interviews.

We rewatched all videos and selected informative video clips that could reflect Ms. Wu’s CK and PK. The first author transcribed the selective video clips manually on her own. Before embarking on transcription for this project, she was trained in classroom video transcription. She had already transcribed classroom videos sufficiently, demonstrating high precision in translating video into text. The recorded interviews were also transcribed with utmost care and precision.

Step 2. Review and labeling of relevant information.

We carefully reviewed the transcriptions of the videos and interviews and highlighted the text related to CK in yellow and underlined the text related to PK.

Step 3. Identification of CK and PK indicators.

According to the CK and PK frameworks, we identified the CK and PK indicators in the transcriptions. We examined the CK and PK present in several videos and segments of interviews to guarantee the reliability of CK and PK extraction. After reaching 90% accuracy, the first author identified the CK and PK indicators involved in the rest of the videos and interviews.

#### Lesson plan analysis

The lesson plans were used for analyzing the teacher’s CK and PK. Together, we first read through the 12 lesson plans and labeled vital information related to the research questions. Collaboratively, we proceeded to identify the CK and PK indicators involved in the 12 lesson plans according to the CK and PK frameworks.

### Ethical and validity issues

This study was conducted with the ethical approval from the Human Research Ethics Committee (HREC), the authors’ university (Reference No. 2021-2022-0334). A letter outlining the research and consent forms was provided to the kindergarten principal and Ms. Wu, and permission was obtained from them. Since the children in this study were 5–6 years old, letters and consent forms were also provided to parents/guardians through kindergarten.

The findings were validated through data triangulation, member checking and inquiry auditing (Creswell, 2014). We collected data from multiple sources for triangulation. Member checking was conducted by re-interviewing Ms. Wu to ensure that her ideas stayed in line with her previous interview responses and the researchers’ interpretations. In addition, two senior researchers in ECE acted as the auditors to ensure the rigor of the research procedure and confirm that the findings appropriately reflected important aspects of the observations, interviews, and lesson plans.

## Findings

### CT concepts, practices, and perspectives taught by the teacher

Our evidence revealed that Ms. Wu emphasized CT concepts across her programming and CT teaching instead of CT practices and CT perspectives (see Table 3).

**Table 3:** Frequency of each CT skill in different data.

CT dimensions	CT components	Videos	Interviews	Lesson plans
CT concepts	Sequences	12 (100%)	12 (100%)	12 (100%)
	Loops	10 (83.3%)	10 (83.3%)	10 (83.3%)
	Conditionals	10 (83.3%)	10 (83.3%)	10 (83.3%)
	Events	12 (100%)	0	0
	Representation	12 (100%)	0	0
	Hardware/ Software	0	0	0
CT practices	Algorithmic design	12 (100%)	0	0
	Pattern recognition	10 (83.3%)	0	0
	Abstraction	0	0	0
	Debugging	0	0	0
	Decomposition	0	0	0
	Iteration	0	0	0
	Generalizing	0	0	0
CT perspectives	Expressing and creating	5 (41.7%)	5 (41.7%)	5 (41.7%)
	Connecting	12 (100%)	12 (100%)	12 (100%)
	Perseverance	3 (25%)	0	0
	Choices of conduct	0	0	0

a: The notation “12 (100%)” indicates that the CT skill was present in all 12 PCT activities with a frequency of 100%. Similarly, “10 (83.3%)” indicates that the CT skill was present in 10 activities with a frequency of 83.3%, and so on. b: During the interview, the 12 unplugged programming lesson plans were presented to the teacher, who was asked to identify the core content covered in each activity. The frequency of each CT skill was then calculated based on the teacher’s responses.

#### CT concepts

Our analysis found that Ms. Wu primarily focused on teaching CT concepts, particularly sequences, loops, and conditionals. These concepts were systematically integrated into her lessons, with sequencing introduced in K1, conditionals and loops introduced in K2 and further developed in K3.

While explicit instruction in the concepts of representation and events was absent from her teaching practices and lesson plans, children learned them through activities such as using Programming Blocks to represent routes and experiencing the correspondence between actions and instructions. The concept of hardware/software was not covered due to the constraints imposed by the unplugged programming tool.

#### CT practices

The video data analysis showed a clear focus on algorithmic design and pattern recognition in the teaching of programming and CT. Algorithmic design was manifested in the development of routes, while pattern recognition was observed in creating repeated routes. However, neither of these terms was explicitly referenced during the interviews nor present within the lesson plans.

**Figure 3:**
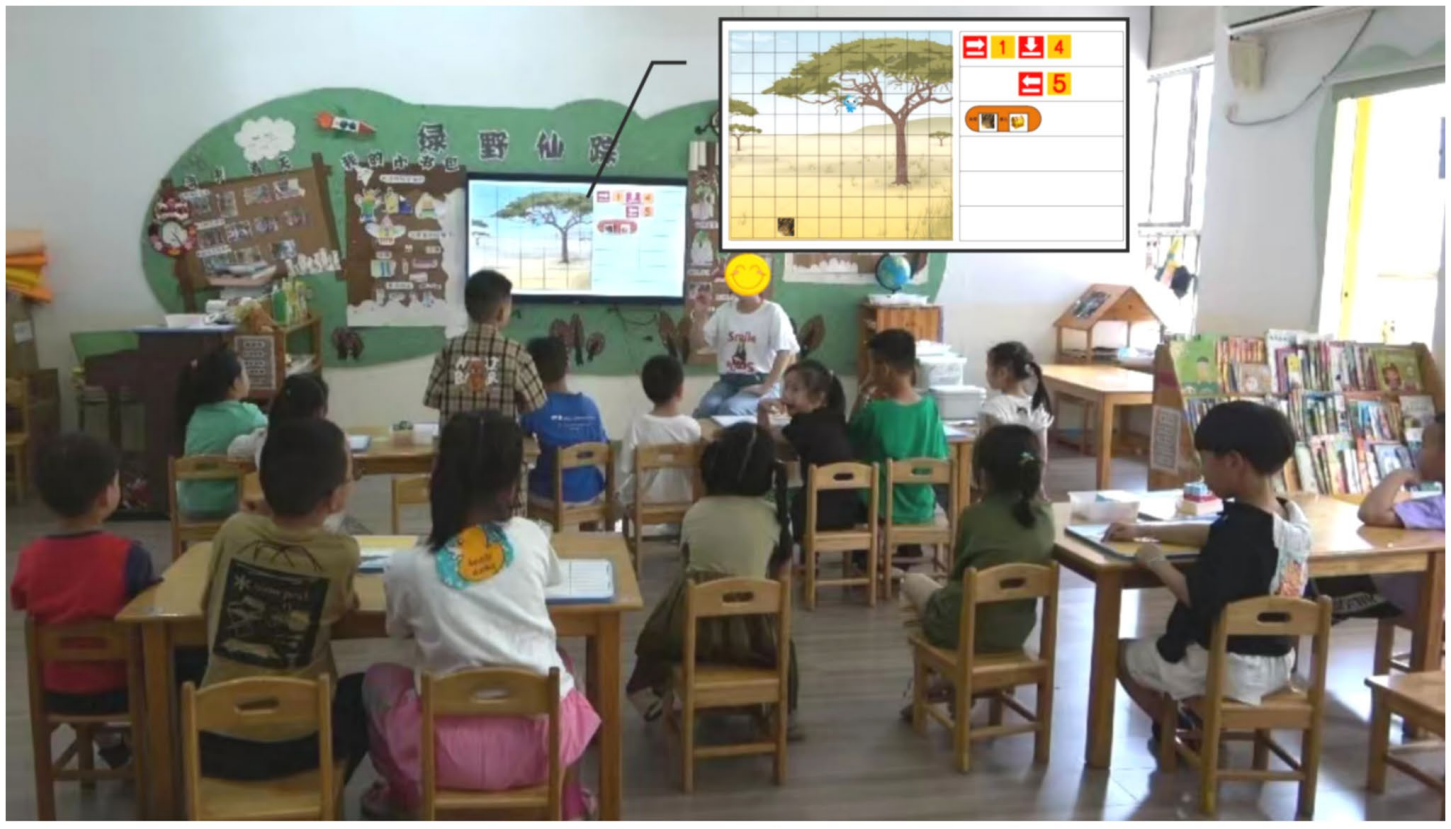
Ms. Wu Presented the “Backward Reasoning Task” with PPT.

The teaching of other CT practices, including debugging, decomposition, abstraction, iteration, and generalizing, was neither evident in Ms. Wu’s teaching practices nor present in the lesson plans. An example involved Ms. Wu’s observation of an erroneous program created by a child. Instead of guiding the child to observe and identify the error, Ms. Wu worked with the child to remove the programming blocks from the programming area and let the child recreate the programs. This approach missed the opportunity to teach debugging skills to the child. Another instance where an opportunity for teaching decomposition emerged was during the “Backward Reasoning Task.” This task necessitated children to complete a path based on information in the programming area and grid map. Although the task provided an opportunity to teach decomposition (see Figure 3), Ms. Wu did not introduce this skill. Additionally, these CT practices were not mentioned by Ms. Wu in the interview. When asked about the core content of early programming and CT education, as well as what children can learn from tasks such as “Backward Reasoning Task,” Ms. Wu did not reference these CT practices.

#### CT perspectives

Ms. Wu displayed an awareness of cultivating children’s CT perspectives of creating and connecting; however, she did not give these aspects prominence in her instructional practices. Five activities designed by Ms. Wu involved fostering children’s creativity; however, these primarily centered on encouraging children to design various routes to enhance their creativity, without affording them opportunities to apply their programming and CT skills in creating projects or expressing ideas, which could better cultivate children’s creativity. In fostering connections among children, Ms. Wu employed pair programming; nonetheless, during pair programming, her focus was primarily directed toward checking the accuracy of the children’s devised routes, while aspects of observing and facilitating collaboration received limited attention.

Moreover, she did not intentionally develop the children’s persistence and choices of conduct. Throughout the 12 sessions, Ms. Wu showed concern for children’s persistence only on three occasions; and she did not mention persistence in her interview or lesson plans. Furthermore, while Ms. Wu underscored the importance of cultivating positive behaviors among children, her approach primarily relied upon issuing directives and reminders, rather than empowering children to make autonomous decisions.

### Pedagogies employed by the teacher

Ms. Wu employed group activities to teach programming and CT with highly structured, task-based activities (see Table 4). She integrated CT skills into tasks that gradually increased in difficulty and guided children to learn CT skills by completing these tasks. Ms. Wu provided ample time and support for the children’s self-exploration and acted as a facilitator and collaborator rather than an authority figure.

**Table 4:** The pedagogical steps of a programming activity.

Time	Steps	Purpose of each step
1–2 min	The teacher begins by telling a story and setting up a scenario for the activity.	To pique children’s curiosity and engage their interest
10 min or so	The teacher introduces Task 1 and teaches the key concepts through its completion by the children.	To instruct the core CT skills to the entire group
30 min or so	The children work in pairs to complete Task 2 and/or Task 3 and share their completed work with the class.	To allow adequate time for children to practice, assess their work, offer prompt feedback, and explore common difficulties during the sharing session
1–2 min	The children tidy up the programming materials.	To cultivate positive habits in children

Ms. Wu utilized a range of pedagogical strategies to support children’s programming and CT learning. Our analysis identified eight strategies, five of which were effective: the unplugged approach, contextualization, embodied cognition, external memory support scaffolding, and a step-by-step strategy for teaching loops. Ms. Wu embraced the unplugged approach to teaching programming and CT, which involves screen-free and hands-on activities. Additionally, she employed contextualization to provide meaningful contexts for programming and CT learning, such as integrating loops learning into the narrative of aiding Qiqi in exploring planetary mysteries. Furthermore, she utilized embodied cognition. The children interacted with an external surrogate named Qiqi, manipulating it to navigate a grid map based on provided instructions. They also physically moved within the grid on the floor, following the given directives. External memory support scaffolding was another strategy Ms. Wu employed, such as using visible Programming Blocks for notating children’s algorithms to support their thinking and problem-solving. Furthermore, she implemented a step-by-step teaching strategy, drawn from early mathematics, to effectively teach loops.

However, the strategies of demonstration, pair programming, and differentiated instruction were not effectively utilized. While Ms. Wu often used demonstrations to exhibit how to identify coordinates, verify routes, and organize materials, she did not model problem-solving skills like debugging and decomposition, nor did she exemplify cooperation and a positive attitude toward mistakes. In addition, Ms. Wu employed pair programming, wherein two children with neighboring school numbers collaborated on programming tasks. However, observations indicated that Ms. Wu did not intentionally and actively observe and facilitate children’s collaboration. Consequently, pair programming proved ineffective, frequently resulting in a lack of genuine interaction and cooperation between the two children, or in some instances, one child would assume a dominant role while the other remained disengaged. Furthermore, Ms. Wu implemented differentiated instruction after recognizing that less capable children struggled to keep up and remained less engaged during programming and CT activities. However, her approach simply involved segregating children into two groups based on their abilities, slowing down the teaching pace, removing challenging tasks for the less capable group, and failing to provide targeted scaffolding for these children to grasp programming and CT concepts.

## Discussion

This study represents a groundbreaking endeavor to investigate an early childhood teacher’s CK and PK in teaching young children programming and CT. Video analysis revealed that Ms. Wu did the most intentional and systemic teaching in CT concepts. However, it was observed that she missed opportunities to expose children to CT practices (e.g., decomposition, debugging) and CT perspectives (e.g., perseverance, choices of conduct). This finding suggests that Ms. Wu possessed a robust foundation of knowledge regarding CT concepts but had limited knowledge of CT practices and perspectives. This conclusion was also supported by evidence from the interviews and lesson plans. Due to Ms. Wu’s lack of clarity regarding the core CT practices and perspectives that children should learn, she did not intentionally integrate CT practices and perspectives into her teaching. As stated by Zhang (2015), if teachers lack an understanding of the diverse CK that should be taught, they will not devote sufficient time and effort to support children’s learning in certain areas. Notably, not only have CT practices and perspectives been neglected in educational practice, but there is also a lack of intervention studies on CT practices and perspectives. A literature review conducted by Lye and Koh (2014) on intervention studies revealed that the majority of studies (85%) solely focused on examining learning outcomes related to CT concepts, with only a small fraction (8 studies) reporting on CT practices or perspectives.

In terms of the learning context, researchers indicated that programming and CT are everywhere in children’s lives; integrating programming and CT into their daily routines and tasks, such as brushing teeth, washing hands, or making objects with clay, offers meaningful learning contexts (Lee and Junoh, 2019; Mills et al., 2021). However, according to interviews, Ms. Wu solely taught programming and CT through whole-group activities and was unaware of the learning opportunities present in daily activities and other learning domains. This can be attributed to Ms. Wu’s limited CK in CT practices and perspectives. As noted by Zhou et al. (2006), teachers who possess strong CK can effectively support children’s learning in any context.

Regarding activity structure and pedagogical approaches, it was found that Ms. Wu created a highly structured and task-based approach by carefully preparing materials and planning activities. This approach enabled Ms. Wu to offer substantial support for children’s programming and CT learning, keeping them engaged in the assigned tasks. The significance of teacher scaffolding in facilitating children’s programming and CT learning has also been highlighted by Newhouse et al. (2017) and Wang et al. (2020). They emphasized that without teachers’ guidance, students are prone to losing interest in programming activities and are unlikely to demonstrate any actions that could be associated with an understanding of “algorithms.” However, it is worth noting that while this approach allows teachers to provide sufficient guidance and support for young children, it may not foster their autonomy and creativity (Kokotsaki et al., 2016).

This study identified eight pedagogical strategies Ms. Wu employed to support children’s programming and CT learning. Among these, five were notably effective, while three showed limited effectiveness. Further analysis suggests that Ms. Wu’s effective utilization of these strategies stems from her consideration of children’s general learning characteristics. The unplugged strategy and embodied cognition align with the hands-on learning style commonly observed in young children (Macrine and Fugate, 2022). Similarly, the contextualization strategy hinges on the widely recognized principle that children learn best when presented within engaging or authentic contexts that capture their interest (Perin, 2011). Additionally, the application of external memory support scaffolding corresponds with the well-established understanding that young children possess limited working memory capacity (Macrides et al., 2021). Only the step-by-step strategy for teaching loops considers the developmental trajectory of children when learning loops; however, according to Ms. Wu, this strategy was transferred from the mathematical domain.

Regarding the less effectively utilized teaching strategies, we found that their successful implementation demands a solid grasp of CK or children’s developmental knowledge within the programming and CT domain. Effective demonstration, for instance, necessitates that teachers possess a strong understanding of the content within the programming and CT domains. This understanding enables them to precisely determine what aspects to model for young children and where to place emphasis during the modeling process. Similarly, successful pair programming requires sensitivity to the core content of “connecting” and proactive observation and intervention to facilitate children’s productive collaboration in programming and CT learning. Additionally, differentiated instruction relies on teachers’ awareness of children’s developmental trajectory in programming and CT to provide tailored scaffolding.

These findings indicated that Ms. Wu exhibited proficiency in applying general pedagogical knowledge (GPK) to programming and CT education but was weak in utilizing content-specific pedagogical knowledge (CPK), which necessitates a solid understanding of the CK in programming and CT education. This finding supported the PCK theory, which Shulman introduced to emphasize the fundamental role of subject matter in teaching (Shulman, 1986). Ball and McDiarmid (1989) also pointed out that teachers with a deeper understanding of the teaching content were more likely to use effective pedagogical strategies to enhance students’ understanding of the subject matter. Moreover, Strawhacker et al. (2018) and Wang et al. (2020) also found that teachers with a stronger CK were better equipped to provide explicit scaffolding in programming and CT education.

Furthermore, the kindergarten in this study used an unplugged approach to teaching programming and CT. They developed an unplugged coding set consisting of three components – the object being programmed, programming tasks, and programming blocks. This board game coding set allows children to learn programming and CT. It is simple to reproduce, as it can be made using basic materials by following the steps provided in Appendix 3.

## Limitations and implications

### Limitations

Although this study is the first to examine an early childhood teacher’s CK and PK for early programming and CT, it does come with certain limitations. Firstly, this study was based on a single teacher as a case study. While this chosen case has provided insights into the teaching of programming and CT within the context of Chinese early childhood education, caution should be exercised when generalizing the findings to broader contexts or other educators. Secondly, this study solely focused on the early childhood teachers’ CK and PK of programming and CT, without investigating the teacher’s knowledge of students, which is a crucial component of teachers’ PCK. Future studies should also explore early childhood teachers’ understanding of students’ learning in programming and CT.

### Practical implications

This study has important implications for practice. CT encompasses more than just CT concepts; it also involves CT practices and perspectives (Brennan and Resnick, 2012). When introducing CT in ECE settings, the goal is not simply to teach young children to “Learn to Code” but rather to equip them with problem-solving skills and attitudes that can be applied beyond programming (Lye and Koh, 2014). CT practices and perspectives are exactly related to problem-solving skills and attitudes. However, it was found that the case teacher’s intentional teaching in CT practices and perspectives was limited, and her knowledge of CT practices and perspectives was weak. Thus, it is crucial to provide teachers with professional support to help them understand the goal of programming and CT education and to enhance their knowledge of CT practices and perspectives. This will help teachers move from a focus on teaching CT concepts to intentionally integrating CT practices and perspectives into their teaching practices.

Furthermore, Ms. Wu’s teaching approach was limited to whole-group activities. She lacked awareness of providing opportunities for children to learn and apply programming and CT in their daily lives. By developing a clear goal for programming and CT education and acquiring a strong CK in CT practices and perspectives, teachers can become equipped with the awareness, knowledge, and ability to integrate programming and CT into children’s daily lives. This inclusive approach ensures that programming and CT skills are accessible to all children, particularly those from disadvantaged backgrounds.

Additionally, there are various pedagogical approaches for teaching early programming and CT, ranging from a task-based approach, where learning activities are centered around tasks guided by adults (McCormick and Hall, 2021), to project-based learning, which emphasizes student-centeredness (Kokotsaki et al., 2016). However, Ms. Wu solely employed a highly structured task-based approach, which prioritized guidance but overlooked students’ autonomy and creativity. Therefore, teachers should adopt a flexible way by combining different pedagogical approaches to teach programming and CT. This enables the provision of necessary guidance while also encouraging students’ autonomy and creativity.

Lastly, based on the unplugged programming tool developed by the case kindergarten, a guide for crafting an unplugged coding set has been innovatively proposed. Programming tools play a crucial role in implementing programming and CT education. This age-appropriate, board game-like coding set extends young children the opportunity to engage in programming and CT activities within both formal and informal settings. Additionally, this unplugged coding set boasts ease of reproduction, as it can be made following straightforward steps and utilizing readily available materials.

### Research implications

This study makes an important contribution to the research. The constructed PK framework for programming and CT, based on an extensive literature analysis, provides a useful tool for analyzing teachers’ PK in teaching programming and CT. In addition, the study presents a model case showcasing the application of CK and PK frameworks to investigate teachers’ CK and PK in early programming and CT education.

Moreover, this study found that teachers had limited CK in CT practices and perspectives and insufficient content-specific pedagogical knowledge (CPK). Therefore, further research could explore ways to enhance teachers’ pedagogical content knowledge in programming and CT education through training programs. It is especially important to investigate how teachers can effectively apply the acquired CK and CPK to their own teaching context to facilitate the integration of programming and CT into classrooms. Previous studies have demonstrated that coaching is critical in facilitating the transfer of training content to teachers’ specific teaching situations (Neuman and Cunningham, 2009). Hence, future researchers could explore transferring the coaching model to the Chinese cultural context to enhance teachers’ intentional and effective teaching of programming and CT.

## Author’s note

This work represents an accomplishment of the Research Project (2024) of the Zhejiang Federation of Humanities and Social Sciences.

## Data availability statement

The raw data supporting the conclusions of this article will be made available by the authors, without undue reservation.

## Ethics statement

The studies involving humans were approved by the Human Research Ethics Committee (HREC), The Education University of Hong Kong. The studies were conducted in accordance with the local legislation and institutional requirements. The participants provided their written informed consent to participate in this study. Written informed consent was obtained from the individual(s) for the publication of any potentially identifiable images or data included in this article.

## Author contributions

YZ: Conceptualization, Methodology, Data collection, Data analysis, Writing – Original draft, and Writing – Review and Editing. WY: Data analysis, Writing – Review & Editing, and Supervision. AB: Review & Editing, and Supervision. All authors contributed to the article and approved the submitted version.
