# Corrigendum: Teaching programming and computational thinking in early childhood education: a case study of content knowledge and pedagogical knowledge

**DOI:** 10.3389/fpsyg.2023.1342297

**Published:** 2023-12-12

**Authors:** Yue Zeng, Weipeng Yang, Alfredo Bautista

**Affiliations:** ^1^School of Education, Wenzhou University, Wenzhou, China; ^2^Department of Early Childhood Education, The Education University of Hong Kong, Hong Kong, Hong Kong SAR, China

**Keywords:** programming, computational thinking, early childhood teacher, content knowledge, pedagogical knowledge

In the published article, there was an error in the affiliation list. For funding purposes, the affiliation “School of Education, Wenzhou University, Wenzhou, China” should be listed as the first affiliation.

The authors apologize for this error and state that this does not change the scientific conclusions of the article in any way. The original article has been updated.

