# Supplementary material for: Teaching programming and computational thinking in early childhood education: a case study of content knowledge and pedagogical knowledge
Source: Front Psychol. 2023 Oct 2;14:1252718. doi: 10.3389/fpsyg.2023.1252718 (PMC10577223; doi:10.3389/fpsyg.2023.1252718)
Supplement: Supplementary file 1 [file Data_Sheet_1.docx]

# **Appendixes**

# **Appendix 1**

*Examples of Data Analysis*

| **Data Types** | **Transcripts** | **CK indicators involved** | **PK indicators involved** | | | |
| --- | --- | --- | --- | --- | --- | --- |
|  |  |  | **Teaching context** | **Activity structure** | **Pedagogical approaches** | **Pedagogical strategies** |
| **Video data** | Teacher: Today, Qiqi will take a spaceship to reach the Moon, Jupiter and Uranus to explore the mysteries of the three planets. Qiqi wants to go to the Moon first. [Contextualization] Do you know where the Moon is located?  Children: Row 5, column 7.  Teacher: Qiqi needs to take a route with loops to reach the Moon. Have you found a route with loops? [Loops]  (Child 1 raises his hand)  Teacher: Yes, please share your idea.  Child 1: One step forward, one step to the left, one step forward, one step to the left, one step forward, one step to the left (Child 1 describes the route while gesturing with his hand) (The teacher notes down the route described by Child 1 on the board using arrows).[External memory support scaffolding] [Representation]  Teacher: Let's move our fingers along the route XXX described and see if it's correct [Embodied cognition]  … | Loops,  Representation | Group activity | Highly structured | Task-based learning | Contextualization,  External memory support scaffolding,  Embodied cognition |
| **Interview data** | Interviewer: What do you consider the core content of early programming and CT, or what do you include in your unplugged programming curriculum?  Teacher: In the first semester of our unplugged programming curriculum, children learned how to use programming blocks to give instructions such as “go forward” “go backward” “go left” and “go right” through floor games. [Representation] [Embodied cognition] In the second semester, in addition to learning how to give instructions of walking in different directions, children also learned how to give instructions for walking several steps in different directions. [Sequences] In the K2 class, we introduce board games as a medium for learning. [Embodied cognition]Children also need to learn about conditionals and loops. [Conditionals and Loops] In the K3 class, the routes children need to program are longer and more complex [Sequences] compared to the K2 classes. Children learn to use a variety of instructions for sequences, conditionals, and loops in a single route. [Sequences, Conditionals, and Loops] They also design different tools on blank Tool Blocks [Expressing and creating] to help Qiqi solve problems.  … | Representation,  Sequences,  Loops,  Conditionals,  Expressing and creating |  |  |  | Embodied cognition |
| **Lesson plan** | **Activity 2: Exploring the Planets**  **Learning Objectives**  1. To use the Loops Blocks independently and use the correct Number Blocks and Directional Blocks to solve problems. [Sequences,  Loops]  2. To experience the joy of cooperative programming. [Connecting]  **Learning Preparation**  1. Scenario Blocks: Moon Block, Jupiter Block, Uranus Block, Meteorite Blocks.  2. Programming Blocks: Directional Blocks, Number Blocks, Loops Block.  3. The Outer Space Board.  4. PPT.  **Learning process**  **1. Create a situation of going to planets to explore their mysteries.** [Contextualization]  --Do you remember Qiqi’s dream? (PPT: outer space)  -- What equipment does Qiqi need to take with him to explore outer space? (PPT: spacesuit, oxygen kit, and translator)  --With these equipments, Qiqi can take a spaceship to explore outer space! Qiqi wants to go to the Moon, Jupiter, and Uranus to explore their mysteries! (PPT: the Moon, Jupiter, and Uranus)  **2. Design routes with loops to the Moon**  --Qiqi plans to go to the Moon first.  --Qiqi has to take a route with loops to reach the Moon. Have you found a route with loops? [Sequences, Loops]  (Ask several children to answer)  ---You designed different routes with loops to help Qiqi reach the Moon. What is the mystery of the Moon? Let’s listen to it. (PPT: the mystery of the Moon)  **3. Design routes with loops to Jupiter and Uranus**  -- What are the mysteries of Jupiter and Uranus? Do you want to know?  -- We have to find these two planets first. Do you know where the two planets are located??  -- Again, Qiqi has to take routes with loops to reach Jupiter and Uranus. Can you help Qiqi design different routes with loops? [Sequences, Loops]  --There are many meteorites in outer space. Remember to go around them! [Algorithmic design]  (Children using the unplugged coding set in pairs [Pair programming] to design routes with loops to Jupiter and Uranus while the teacher goes around to check and guide them.)  (Children share their looping routes to Jupiter and Uranus.)  --You designed different routes with loops to help Qiqi reach Jupiter and Uranus. What are the mysteries of Jupiter and Uranus? Let us listen to it. (PPT: the mysteries of Jupiter and Uranus)  --With your help, Qiqi has reached the Moon, Jupiter and Uranus. Where else will Qiqi go on the spaceship? See you next time. | Sequences,  Loops,  Algorithmic design,  Connecting | Group activity | Highly structured | Task-based learning | Contextualization,  Pair programming |

# **Appendix 2**

**Interview Protocol: Teachers’ Content Knowledge and Pedagogical Knowledge in Early Programming and CT**

**Before the interview**

Thank you very much for allowing me to observe and videotape your classes this semester and for taking the time to be interviewed. I want to ask you some questions based on my observed activities. Since your answers are important to my research, I would like to record our conversation, okay?

***Start the audio recording with the consent of the interviewee***

***(The following questions are only the outline of the interview, and the actual interview will be flexible according to the teacher’s answers)***

**Part A Basic Information**

1. Could you briefly introduce yourself, including your age, education, working experience, etc.?
2. How many years of early childhood education experience do you have in total (excluding years of study)?
3. How long have you taught programming and CT?

**Part B Content Knowledge**

1. What do you think is the core content of early programming and CT?
2. There are 12 programming and CT activities for this semester, and here are the lesson plans for these 12 activities (show the lesson plans). Can you tell me the core content covered in each activity?
3. (If “decomposition” is not mentioned) What could the children learn from the “Backward Inference Task”?
4. How do you understand XXX (XXX stands for the core content mentioned by Ms. Wu)?

**Part C Pedagogical Knowledge**

1. What materials did you provide to help children learn programming and CT? Why did you provide these materials?
2. Have you conducted other forms of programming and CT activities besides group activities (such as integrating programming and CT into the learning center, children’s daily routines or other learning domains?)
   - 1. If yes, how?
     2. If no, do you have any ideas about how to integrate?
3. What is the basic process of the programming and CT group activities?
4. What pedagogical approaches did you employ (e.g., task-based learning, play-based learning, project-based learning)? Why did you employ this approach?
5. What pedagogical strategies did you use in teaching programming and CT?
6. Why did you use XXX (XXX stands for the pedagogical strategies mentioned by Ms. Wu)?
7. What do you think you did and did not do well in supporting young children to learn programming and CT? Why?

**Part D Final Question of the Interview**

1. Do you have any further comments?

# **Appendix 3**

**Steps for Making an Unplugged, Boardgame-Like Coding Set**

**Step 1:** Create the object to be programmed. Cut out a card (being careful that the card size does not exceed the size of the grid on the board) and draw a pawn on the card, or use a toy as the object to be programmed.

**Step 2:** Make a grid map for the pawn to move. Take a large piece of paper and draw grids on it, for example, 10 by 10 grids.

**Step 3:** Create chess pieces for programming tasks. Cut some cards (being careful that the size of the cards is at most the size of the grid on the board) and draw places and tools that appear in the programming tasks on them. Use your imagination to create fun scenarios. When playing, place these cards on the grid map according to the designed programming task.

**Step 4:** Make programming cards. Cut some cards and write numbers, arrows, and patterns that represent loops and conditionals on them to make number cards, directional cards, loops cards, and conditional instruction cards (see Figure 2).

Then you can play the board game with your friends! One person designs a programming task, one "writes" instructions by placing programming cards on the paper or floor, and one moves the pawn on the grid map to verify the instructions. You can also make up other rules to make the game more enjoyable!
